# Telephone-delivered psychotherapy for rural-dwelling older adults with generalized anxiety disorder: study protocol of a randomized controlled trial

**DOI:** 10.1186/1471-244X-14-34

**Published:** 2014-02-08

**Authors:** Gretchen A Brenes, Suzanne C Danhauer, Mary F Lyles, Michael E Miller

**Affiliations:** 1 Department of Psychiatry and Behavioral Medicine, Wake Forest School of Medicine, Medical Center Blvd., Winston-Salem, NC 27157, USA; 2 Department of Social Sciences and Health Policy, Wake Forest School of Medicine, Medical Center Blvd., Winston-Salem, NC 27157, USA; 3 Department of Geriatrics, Wake Forest School of Medicine, Medical Center Blvd., Winston-Salem, NC 27157, USA; 4 Department of Biostatistics, Wake Forest School of Medicine, Medical Center Blvd., Winston-Salem, NC 27157, USA

**Keywords:** Generalized anxiety disorder, Older adults, Randomized controlled trial, Rural mental health, Telephone-delivered psychotherapy

## Abstract

**Background:**

Generalized Anxiety Disorder (GAD), characterized by excessive and uncontrollable worry, has a negative impact on the health, well-being, and functioning of older adults. Cognitive behavioral therapy has demonstrated efficacy in reducing anxiety and worry in older adults, but the generalizability of these findings to community-dwelling older adults is unknown. The aim of the current study is to examine the efficacy of a cognitive-behavioral intervention delivered by telephone in reducing anxiety and worry in rural community-dwelling older adults with GAD.

**Methods/Design:**

We propose a randomized controlled trial comparing telephone-delivered cognitive behavioral therapy (CBT-T) with nondirective supportive therapy (NST-T). One hundred seventy six adults 60 years and older diagnosed with GAD will be randomized to one of the two treatment conditions. The primary outcomes are self-report worry and clinician-rated anxiety. Secondary outcomes include depressive symptoms, sleep, quality of life, and functional status.

**Discussion:**

It is hypothesized that CBT-T will be superior to NST-T in reducing anxiety and worry among older adults with GAD. Further, CBT-T is hypothesized to be superior to NST-T in reducing problems with depressive symptoms, sleep, functional status and quality of life. If this program is successful, it could be implemented as a low-cost program to treat late-life anxiety, especially in rural areas or in circumstances where older adults may not have access to qualified mental health providers.

**Trial registration:**

clinicaltrials.gov Identifier: NCT01259596

## Background

Anxiety disorders are the most prevalent psychiatric disorders [1]. Among older adults, anxiety is more common than depression [2]; yet research on the nature and treatment of anxiety has lagged far behind that of depression [3]. Generalized Anxiety Disorder (GAD) is characterized by excessive, uncontrollable worry and somatic symptoms and is one of the most common anxiety disorders in older adults, with prevalence rates in community-dwelling older adults ranging up to 7.3% [4]. The effects of GAD extend beyond mental health. GAD is associated with significant economic burden [5], poorer quality of life [6], increased comorbidity and disease burden [7], sleep disturbances [8], and increased health services use [9].

Cognitive behavioral therapy (CBT) is the most efficacious nonpharmacological treatment for GAD [10] and has demonstrated efficacy in the treatment of late-life GAD [11]. However, limitations of these studies may reduce the generalizability of their findings. Most importantly, attrition rates are high (up to 33%) which may reflect dissatisfaction or difficulty overcoming barriers, such as transportation, financial difficulties, and stigma. Additionally, most studies have been conducted in academic settings and required up to 15 face-to-face sessions. Treatment has often been provided in a group format, producing even more constraints. Anxious older adults face many barriers to treatment that have not been addressed in prior studies. Therefore, it is not known if previous findings will generalize to community-dwelling older adults. Studies are needed that apply to community-dwelling elders, both by reducing their barriers to treatment and increasing their access to treatment.

Conducting treatment using telephone therapy and written materials reduces barriers that older adults face, including stigma associated with mental health treatment, as treatment can be conducted within the privacy of one’s home. No face-to-face visits are required, eliminating potential transportation barriers. Bibliotherapy with telephone therapy also increases the accessibility to mental health services for the many people who live in underserved areas and lack access to a trained geriatric cognitive-behavioral therapist. This format is particularly appropriate for older adults because there are fewer time constraints. In a typical CBT intervention, patients attend 8 to 16 weekly 50-minute psychotherapy sessions. Participants are taught CBT principles and techniques and are instructed on how to apply them to their daily lives. Therefore, sessions must be very structured to complete the treatment. However, older adults tend to need more time to process information in psychotherapy, especially since those with GAD often have impairments in short-term memory [12]. Thus, an intervention that provides a variety of approaches and additional time to process information may be particularly beneficial for older adults. The combination of telephone therapy and written materials allows older adults to process information both visually and verbally. The written format provides older adults with an easy opportunity to read and review the materials as often as needed and at their own pace. In our pilot study, older adults report that they frequently reread and highlight information [13]. By presenting written information beforehand, the telephone session can be used to briefly review materials and answer questions and then place a greater emphasis on the application of anxiety management techniques in the participant’s daily life.

### Aims and hypotheses

The primary aim of this study is to compare the efficacy of telephone-delivered cognitive-behavioral therapy (CBT-T) with nondirective supportive therapy (NST-T; an active comparison condition) for reducing anxiety and worry. We hypothesize that CBT-T will be superior to NST-T in reducing anxiety and worry. Our secondary hypotheses are that CBT-T will be superior to NST-T in improving coexistent symptoms (depressive symptoms and sleep) and functional status (disability and quality of life). The hypotheses will be tested on outcome measures assessed immediately upon completion of the intervention (i.e., the 4-month post-randomization visit).

## Methods/Design

### Study design

**Figure 1:**
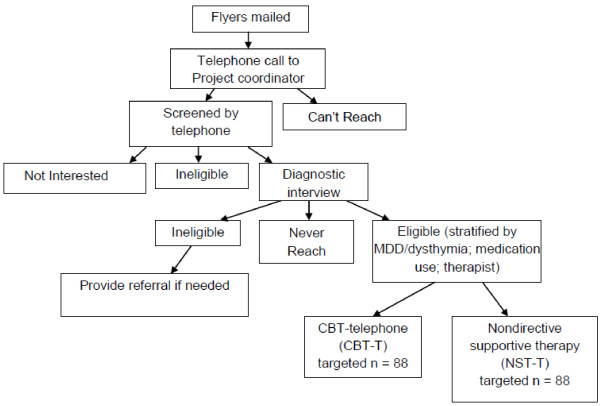
CONSORT flow diagram.

The study design is a single-site randomized controlled trial comparing telephone-delivered cognitive behavioral therapy (CBT-T) with nondirective supportive therapy (NST-T) for the treatment of late-life GAD in a sample of rural-dwelling older adults (Figure 1). Participants will be recruited from the 41 counties in North Carolina with an urban population of <20,000 people. CBT-T consists of up to 10 workbook chapters accompanied by 8 to 12 weekly 45–50 minute psychotherapeutic telephone calls. NST-T consists of 10 weekly 45–50 minute nondirective supportive therapy telephone calls for 10 weeks. Upon completion of the weekly sessions, all participants will receive an additional 4 telephone booster sessions over the next 3 months. Assessments will be conducted by telephone pre-randomization and at approximately 2 months post-randomization (abbreviated assessment), 4 months post-randomization, 9 months post-randomization, and 15 months post-randomization. Participants will be compensated $25 for each of the pre-randomization, 4-month, 9-month, and 15-month interviews.

### Participants

#### Recruitment

We propose multiple recruitment strategies, including mailing a flyer describing the study to adults aged 60 years and older residing in the targeted recruitment counties using three strategies: (1) a commercial mailing company; (2) mailing letters to local physicians and medical centers, churches, and community agencies, and (3) sending flyers to older adults receiving Meals on Wheels. The recruitment flyer and letters will briefly describe the study and instruct interested persons to call our toll-free telephone number. Using a standardized script, staff will describe the study in greater detail and screen these persons by telephone. After providing verbal consent, Stage 1 of the screening process consists of basic demographic information (age, gender, race/ethnicity, education, county of residence and plans to move within 6 months), hearing impairment, current self-reported worry symptoms, current psychotherapy use, psychotropic medication use and changes in dosage within the last month, current alcohol or drug problems, presence of auditory or visual hallucinations, diagnosis of dementia, general cognitive functioning, and privacy to talk by telephone. Screening will be stopped if any responses indicate study ineligibility. Participants who score ≥ 16 on the worry measure and meet the other inclusion/exclusion criteria will progress to Stage 2 of the screening process where they will complete a second lengthier telephone eligibility interview. During the second interview, the Structured Clinical Interview for the DSM-IV (SCID), an interviewer-rated anxiety measure, and a health services utilization measure will be administered. Participants will also be mailed a battery of self-report measures to complete. Upon completion of both the interview and self-report measures, potential participants will be informed of their eligibility status.

#### Eligibility criteria

Inclusion criteria include: 1) age ≥ 60 years, 2) a principal or co-principal DSM-IV diagnosis of GAD as assessed by the SCID-IV, 3) residency in a county with an urban population of < 20,000 people, and 4) proficiency in English. Principal diagnosis is defined as the disorder with the highest global severity rating. When 2 diagnoses meet this criterion, co-principal diagnoses will be assigned. Exclusion criteria include: 1) current psychotherapy; 2) a DSM-IV diagnosis of active alcohol or substance abuse within the last month; 3) a diagnosis of dementia or global cognitive impairment based on the education-adjusted scores on the Telephone Interview for Cognitive Status-modified (TICS-m); 4) psychotic symptoms assessed by the endorsement of hallucinations or delusions; 5) active suicidal ideation with plan and intent; 6) any change in psychotropic medications within the last 1 month and 7) any hearing loss that would prevent a person from participating in telephone sessions.

#### Randomization

Randomization to therapist and treatment will be stratified on baseline current depression diagnosis (major depression/dysthymia vs. no depression diagnosis) and baseline psychotropic medication use. We will use a permuted block algorithm with random block lengths to generate the randomization assignments. Randomization will be executed via a secure web-based data management system. To ensure masking of the assessment staff to intervention assignment, the randomization procedures will be performed by staff members not involved in the assessments.

#### Sample size calculation

We used data from a pilot study trial comparing CBT-T with usual care with referral for reducing anxiety and worry in older adults with anxiety disorders (N = 55; n = 29 in usual care with referral and n = 26 in CBT-T) [14] to determine the sample size for the current study. Analyses of these pilot data provided estimates of the standard deviation (SD) for the Penn State Worry Questionnaire-A (PSWQ-A) and Hamilton Anxiety Rating Scale (HAM-A) scores: values were 6.7 and 7.2, respectively. The correlation between baseline and post-intervention PSWQ-A scores was 0.45 for the PSWQ-A and 0.40 for the HAM-A. We considered a 0.5 SD difference on the PSWQ-A (3.35 units) and the HAM-A (3.5 units) in the post-intervention means as the minimally important detectable difference. A mixed effects analysis appropriate for repeated measures analysis of covariance will be used to test the primary hypotheses, with a contrast to test the hypothesis of no intervention effect at the post-randomization visit. Using the pilot data estimates of SD and correlation and assuming that each co-primary outcome will be tested at the 0.025 two-sided significance level, power calculations for analysis of covariance on the PSWQ-A outcome indicated the need for a total of 80 participants per group to attain 90% power, 70 participants per group to provide 85% power, and 60 participants per group to attain 78% power. We plan to recruit 88 participants per group to account for an expected drop-out rate of approximately 10%.

### Ethics

This study was approved by Institutional Review Board at Wake Forest School of Medicine.

### Intervention descriptions

#### Cognitive behavioral therapy-telephone (CBT-T)

Telephone therapy. Weekly telephone sessions with the randomly assigned therapist will last 45 to 50 minutes. In order to ensure privacy, every participant will be asked if he/she has adequate privacy for the session, and if not, will be offered an opportunity to reschedule the appointment. All sessions will start with a review of homework and any problems or stressors they have encountered with the homework. The therapist and the participant will discuss whether anxiety coping skills were used and were effective. If so, use of the coping techniques will be reinforced. If the anxiety was not adequately managed, coping skills will be reviewed and ways to incorporate or improve their use will be identified. The therapist will then review the assigned chapter and accompanying exercise with the participant. The participant will be encouraged to ask questions about the reading materials and discuss any difficulties in implementing the anxiety technique. If a participant reports difficulty with a particular chapter or technique, he/she will be instructed to continue to work on that chapter and technique for another week and another call will be scheduled. Upon completion of the workbook, booster sessions will be used to reinforce anxiety management skills and problem-solve issues that arise as needed. Booster sessions will be provided at 2 weeks, 4 weeks, 8 weeks, and 16 weeks after completion of the treatment.

##### Workbook

The principal investigator adapted the 10-chapter workbook from the standardized Controlling Anxiety in Later-Life Medical Patients workbook [15]. The techniques in this CBT intervention have demonstrated efficacy in treating adults with GAD [16,17] and older adults with GAD [18-20]. Chapter 1 describes the treatment and presents a cognitive behavioral model of anxiety. Chapters 2 through 9 each address a specific anxiety management technique or a specific problem that may be comorbid with anxiety. Chapters are approximately 5 to 10 pages long and are written in lay terms. They are presented in a large font, and key points are highlighted and reiterated simply to aid readers in fully understanding the content. Each chapter contains multiple examples of specific situations that an older adult might experience. Chapters are also focused on techniques that are more relevant to older adults, such as sleep and pain, rather than assertiveness skills and time management. The sleep and pain chapters are optional. There are 3 ways to trigger the use of the pain and sleep modules: 1) the participant mentions problems with pain or sleep during the course of the telephone sessions with the therapist; 2) the participant requests the pain and/or sleep modules; 3) the participant scores below the mean (M = 67.5) for older adults on the pain scale from the SF-36 as lower scores indicate greater pain [21] or > 7 on the Insomnia Severity Index indicating at least subthreshold insomnia [22]. Each chapter is followed by a homework exercise to practice the technique described in that chapter. A completed example is provided followed by blank copies to be completed by the participant. The homework is used to encourage the application of the techniques to the participant’s daily life.

#### Nondirective supportive therapy-telephone (NST-T)

Nondirective supportive therapy (the active control group) will follow Borkovec’s protocol [23,24]. NST-T provides a “high-quality therapeutic relationship that provides a warm, genuine, and accepting atmosphere through the use of supportive and reflective communications” (p. 9); it does not provide advice, suggestions, or coping methods. Borkovec reports high levels of treatment credibility and therapeutic relationship [23]. NST-T will be conducted by telephone via weekly 45–50 minute sessions with the randomly assigned therapist. Participants in the NST-T condition will receive 10 sessions. This number was chosen because it is the average number of sessions a participant in the CBT-T condition can receive (minimum of 8 sessions and maximum of 12 sessions). They will also receive 4 booster sessions. The NST-T condition matches the CBT-T condition in terms of amount of therapist contact (equivalent session length, number of sessions, and frequency of sessions) and delivery of treatment (via telephone).

#### Therapist fidelity

At least two therapists will deliver both treatments and supervision for each condition will be conducted weekly. Two experienced doctoral-level clinicians who are otherwise unaffiliated with the study will rate the therapists’ adherence and competency in delivering the psychotherapies.

### Data collection

A combination of interviewer-based and self-report measures will be administered pre-randomization and at approximately 4 months post-randomization, 9 months post-randomization, and 15 months post-randomization. At approximately 2 months post-randomization, participants will also complete an abbreviated battery consisting of the primary outcomes and a depression measure. All interviewer-based measures are administered by telephone by a trained assessor who is blinded to treatment condition. Self-report measures are mailed to the participants with a stamped return envelope enclosed.

#### Diagnostic measure

The Structured Clinical Interview for DSM-IV (SCID) [25] is a semi-structured clinical interview. Only modules for anxiety disorders, depressive disorders, psychoactive substance use disorders, and psychotic disorders (screener only) will be administered.

#### Primary outcome measures

The Hamilton Anxiety Rating Scale (HAM-A) [26] is a 14-item interviewer-rated measure of anxiety symptoms. Ratings are made on a 5-point scale ranging from 0 (none) to 4 (very severe). The Structured Interview Guide for the Hamilton Anxiety Rating Scale will be used in order to increase reliability [27]. It has been validated in samples of older adults with GAD and demonstrates good inter-rater reliability (rs = .81-.85) [20,28,29].

The Penn State Worry Questionnaire-Abbreviated (PSWQ-A) [30,31] is an 8-item measure of the frequency and intensity of worry. Participants rate each item on a 5-point scale and responses are summed, with higher scores indicating greater worry. The full PSWQ has demonstrated reliability and validity in older adults with GAD [18,20,32]. The PSWQ-A has similar internal consistency, better test-retest reliability, and comparable convergent-divergent validity as the full length version [30,33]. The abbreviated version is easier for older adults to complete, because all double negative questions in the original version were eliminated.

#### Secondary outcome measures

The Beck Depression Inventory (BDI-II) [34] is a 21-item measure of depressive symptoms. Responses are summed and can range from 0 to 63. Higher scores indicate greater depressive symptoms. The BDI-II has good psychometric properties in samples of both younger and older adults with GAD [35,36].

The Pepper Assessment Tool for Disability (PAT-D) [37,38] is a 19-item self-report measure of perceived difficulties with mobility and performing basic and advanced activities of daily living during the last month. Participants rate how much difficulty they had performing each of the activities on a 5-point Likert scale ranging from 1 “no difficulty” to 5 “unable to do” the task. This measure has been validated in 4 large samples of older adults and has demonstrated response to change in physical activity interventions [37].

The SF-36 [39] is a self-report measure of health-related quality of life consisting of 36 items that form 8 subscales: physical functioning, role limitations due to physical health problems, role limitations due to emotional health problems, social functioning, freedom from pain, energy or fatigue, emotional well-being, and general health perceptions. The scales range from 0 (maximum impairment) to 100 (no impairment). It has demonstrated good internal validity and construct validity [40,41].

The Insomnia Severity Index (ISI) [22] is 7-item self-report measure of type and severity of insomnia symptoms, including problems with sleep onset, sleep maintenance, or early morning awakening; satisfaction with current sleep pattern; interference with daily functioning; noticing impairment attributed to sleep problems; and level of concern or distress caused by the sleep problem. Participants rate each item on a 5-point scale and responses are summed, with higher scores indicating greater sleep impairment.

#### Crisis protocol

If any participant indicates a significant worsening in anxiety or depression scores defined as a 1 standard deviation decline from baseline or a participant endorses “I would like to kill myself” or “I would kill myself if I had the chance,” the computer software system will automatically generate an e-mail to the PI and the project manager. If there is a need for immediate treatment (e.g., active suicidal ideation, active psychotic symptoms, disorientation, active substance abuse) at any point in time, the staff person will notify the PI. In both cases, the participant may be referred for psychiatric care.

#### Statistical analyses

The focus of the primary analyses will be on the comparisons between the two interventions for each of the primary outcome (HAM-A and PSWQ-A) follow-up measurements. Each co-primary outcome will be tested at the two-sided, 0.025 significance level (i.e. using a Bonferroni adjustment). Comparisons of outcome measures (or transformations thereof) between intervention groups will be made by mixed-model repeated measures analysis of covariance with an unstructured covariance matrix to account for the fact that multiple measurements (at approximately 2 months post-randomization, 4 months post-randomization, 9 months post-randomization, and 15 months post-randomization) from participants are not independent. Each primary hypothesis will be tested at the 4-month visit using a contrast within the framework of this mixed-effects, repeated measures analysis. In the primary analysis, all randomized subjects will be included in their original study group regardless of the final mode of intervention or the extent of compliance with the study protocol; that is, the primary analysis will follow an “intent to treat” philosophy. The analysis of covariance model for the primary outcomes will contain terms for the baseline value of the outcome, therapist (a factor to which participants are randomized), baseline current depressive disorder (used to stratify randomization), use of psychotropic medications at baseline (used to stratify randomization), the intervention effect, a time effect, and the time by intervention interaction term. The interaction term is necessary in the model to allow the test of the primary hypothesis on the 4-month post-intervention measurement. Additional secondary analyses will be performed to test between intervention groups for measurements at approximately 2 months post-randomization, 9 months post-randomization, and 15 months post-randomization. We will also perform a test of interaction between the therapist and intervention effect to explore if the therapist effect moderates the effect of the intervention.

Secondary outcomes, including the BDI-II, PAT-D, SF-36, and ISI will be analyzed using mixed models repeated measures analysis of covariance using techniques like those described for the primary outcomes. We will perform hypothesis tests at the 0.05 level for these outcomes and following the recommendations of Wang et al. [42], we will report the overall probability of Type I error within published manuscripts.

## Discussion

In this RCT, we compare telephone-delivered CBT with NST as a treatment for Generalized Anxiety Disorder in rural older adults. We hypothesize that CBT-T will be superior to NST-T in reducing anxiety and worry. We also hypothesize that CBT-T will be superior in reducing depressive symptoms and sleep problems, and improving functional status.

We have chosen NST as a comparison condition because it provides a structurally equivalent comparison group [43] and has similar levels of outcome expectations and credibility [23,44] as CBT. We have designed the interventions such that all participants get the same number and length of sessions. Thus, NST as a comparison group allows for the demonstration of the superiority of CBT to a structurally equivalent comparison. It also provides all participants with an opportunity to receive treatment.

### Implications for practice

Telephone-delivered psychotherapy is one method of providing treatment to individuals limited by practical barriers. This method may be particularly appropriate for older adults living in rural settings as they are less likely to have access to the internet [45]. Further, patient satisfaction with telephone delivered psychotherapy is high and comparable to face-to-face psychotherapy [14]. Should we demonstrate the efficacy of the CBT-T intervention over NST-T, this low-cost treatment could be implemented widely and could potentially reach large numbers of patients in rural areas, primary care settings, and specialty settings, or in circumstances when older adults have barriers to seeking care.

## Abbreviations

BDI-II: Beck depression inventory; CBT-T: Cognitive behavioral therapy-telephone; GAD: Generalized anxiety disorder; HAM-A: Hamilton anxiety rating scale; ISI: Insomnia severity index; NST-T: Nondirective supportive therapy-telephone; PAT-D: Pepper assessment tool for disability; PSWQ-A: Penn state worry questionnaire-abbreviated; SCID: Structured clinical interview for the dsm-IV; SD: Standard deviation.

## Competing interests

The authors declare they have no competing interests.

## Authors’ contributions

All authors participated in the design of the study. GB is responsible for oversight of the project and data collection. GB and SD are responsible for intervention development and delivery. MM is responsible for database management and data analyses. All authors contributed to the manuscript and approved the final manuscript.

## Pre-publication history

The pre-publication history for this paper can be accessed here:

http://www.biomedcentral.com/1471-244X/14/34/prepub
